# Mutation spectrum and biochemical features in infants with neonatal Dubin-Johnson syndrome

**DOI:** 10.1186/s12887-020-02260-0

**Published:** 2020-08-05

**Authors:** Kwang Yeon Kim, Tae Hyeong Kim, Moon-Woo Seong, Sung Sup Park, Jin Soo Moon, Jae Sung Ko

**Affiliations:** 1 Department of Pediatrics, Seoul National University College of Medicine, 101 Daehak-ro, Jongno-Gu, 110-769 Seoul, Korea; 2 Laboratory Medicine, Seoul National University College of Medicine, Seoul, Korea

**Keywords:** Dubin-Johnson syndrome, Neonatal cholestasis, *ABCC2*, Aspartate transaminase, Alanine transaminase

## Abstract

**Background:**

Dubin-Johnson syndrome (DJS) is an autosomal recessive disorder presenting as isolated direct hyperbilirubinemia.DJS is rarely diagnosed in the neonatal period. The purpose of this study was to clarify the clinical features of neonatal DJS and to analyze the genetic mutation of adenosine triphosphate-binding cassette subfamily C member 2 (*ABCC2*).

**Methods:**

From 2013 to 2018, 135 infants with neonatal cholestasis at Seoul National University Hospital were enrolled. Genetic analysis was performed by neonatal cholestasis gene panel. To clarify the characteristics of neonatal DJS, the clinical and laboratory results of 6 DJS infants and 129 infants with neonatal cholestasis from other causes were compared.

**Results:**

A total of 8 different *ABCC2* variants were identified among the 12 alleles of DJS. The most common variant was p.Arg768Trp (33.4%), followed by p.Arg100Ter (16.8%). Three novel variants were identified (p.Gly693Glu, p.Thr394Arg, and p.Asn718Ser). Aspartate transaminase (AST) and alanine transaminase (ALT) levels were significantly lower in infants with DJS than in infants with neonatal cholestasis from other causes. Direct bilirubin and total bilirubin were significantly higher in the infants with DJS.

**Conclusions:**

We found three novel variants in 6 Korean infants with DJS. When AST and ALT levels are normal in infants with neonatal cholestasis, genetic analysis of *ABCC2* permits an accurate diagnosis.

## Background

Dubin-Johnson syndrome (DJS) is characterized by conjugated hyperbilirubinemia induced by a mutation of the adenosine triphosphate-binding cassette subfamily C member 2 (*ABCC2*) gene. The *ABCC2* gene is located on chromosome 10q24 and contains 32 exons [1, 2]. Mutations in the *ABCC2* gene result in a decrease in the production or loss of function of the Multidrug resistance-associated protein 2 (MRP2) protein, which cannot effectively transport bilirubin out of the hepatocyte. Defects in this transporter inhibit the excretion of bile acids as well as conjugated bilirubin, causing neonatal cholestasis [3]. Aspartate transaminase (AST) and alanine transaminase (ALT) are usually within the normal range, but bilirubin levels fluctuate [1, 4]. The clinical symptoms of neonatal DJS are not obvious, and the syndrome is rarely diagnosed in infants [1]. The purpose of this study was to clarify the clinical features of neonatal DJS and to investigate the mutation of *ABCC2* in Korea.

## Methods

### Comparison of subjects with other infants with neonatal cholestasis

From 2013 to 2018, patients diagnosed with neonatal cholestasis at Seoul National University Hospital were reviewed. A total of 135 patients were enrolled, 6 of whom were diagnosed with neonatal DJS. We defined neonatal cholestasis as serum direct bilirubin level (DB) greater than 1.0 mg/dL when serum total bilirubin (TB) was ≤ 5.0 mg/dL or a serum DB level greater than 20% of serum TB when serum TB was > 5.0 mg/dL [5, 6]. Initial clinical symptoms, laboratory results, liver biopsy findings, and *ABCC2* genetic mutations were reviewed. To clarify the characteristics of neonatal DJS, the clinical and laboratory results of 6 DJS infants and 129 infants with neonatal cholestasis from other causes were compared. Statistical analyses were performed with SPSS version 24.0 (IBM, New York, NY, USA). The Mann-Whitney test was used for comparisons, and a P value of < 0.05 was considered significant. This study was approved by the Institutional Review Board of Seoul National University Hospital (IRB No. 1806-154-953).

### Genetic analysis of ABCC2

The neonatal cholestasis gene panel contained the following 34 genes: *ABCB11, ABCB4, ABCC2, AKR1D1, AMACR, ATP8B1, BAAT, CLDN1, CYP27A1, CYP7A1, CYP7B1, DGUOK, DHCR7, FAH, HSD3B7, JAG1, MPV17, NOTCH2, NPC1, NPC2, NR1H4, PKHD1, POLG, PRKCSH, SERPINA1, SLC10A1, SLC10A2, SLC25A13, SLCO1B1, SLCO1B3, TJP2, TRMU, VIPAS39, VPS33B*. Neonatal cholestasis gene panel testing was performed in infants with suspicious genetic cholestasis including Alagille syndrome, citrin deficiency, DJS, ARC syndrome and progressive familial intrahepatic cholestasis. The construction of pre-capture libraries (Illumina, Inc., San Diego, CA, USA) and capture process (Agilent Technologies, Santa Clara, CA, USA) was performed according to the manufacturer’s protocols. The captured libraries were sequenced using the MiSeqDx (Illumina, Inc., San Diego, CA, USA). Raw sequence data were analyzed the NextGENe software (SoftGenetics, State College, PA, USA) and annotated with ANNOVAR (http://annovar.openbioinformatics.org). The gnomAD (http://gnomad.broadinstitute.org) and KRG (http://coda.nih.go.kr/coda/KRGDB) database were used to filter out the common variants. The ClinVar and Human Gene Mutation Database (HGMD) was used to search the known pathogenic mutations. To predict the functional significance of missense variants in programs predicting, amino acid conservation score (Exome Aggregation Consortium, Polyphen, and MutationTaster) was used. The clinical significance of each variant was classified according to the recent recommendations of the American College of Medical Genetics and Genomics (ACMG) on standards for interpretation and reporting of sequence variations as follows: pathogenic, likely pathogenic, variants of unknown significance (VUS), likely benign, and benign variant [7–10].

## Results

### Diagnosis of neonatal cholestasis

One hundred thirty-five neonatal cholestasis patients were enrolled in the study (Table 1). There were 37 cases (27.4%) of biliary atresia, 11 cases (8.1%) of choledochal cysts, 7 cases (5.2%) of neonatal intrahepatic cholestasis caused by citrin deficiency, 6 cases (4.5%) of neonatal DJS, 6 cases (4.5%) of total parenteral nutrition (TPN) induced neonatal cholestasis and 4 cases (3.0%) of Alagille syndrome. There were 2 cases (1.5%) of arthrogryposis-renal dysfunction-cholestasis syndrome and 2 cases (1.5%) of neonatal cholestasis due to a congenital portosystemic shunt. There was one case each of progressive familial intrahepatic cholestasis and galactosemia (0.7%), and one case each of neonatal cholestasis due to infection, such as herpes simplex virus, cytomegalovirus, and sepsis (0.7%). In 53 cases (39.3%), which was the largest percentage, the cause of neonatal cholestasis was unknown.

**Table 1:** Identified causes of neonatal cholestasis in our cohort

Diagnosis	*N* = 135
Biliary atresia	37 (27.4%)
Choledochal cyst	11 (8.1%)
NICCD	7 (5.2%)
Dubin Johnson syndrome	6 (4.5%)
TPN induced cholestasis	6 (4.5%)
Alagille syndrome	4 (3.0%)
Neonatal hemochromatosis	2 (1.5%)
ARC syndrome	2 (1.5%)
Congenital portosystemic shunt	2 (1.5%)
PFIC	1 (0.7%)
Galactosemia	1 (0.7%)
HSV	1 (0.7%)
CMV	1 (0.7%)
Sepsis	1 (0.7%)
Idiopathic neonatal cholestasis	53 (39.3%)

*NICCD* Neonatal intrahepatic cholestasis caused by citrin deficiency; *TPN* Total parenteral nutrition; *ARC* Arthrogryposis-renal dysfunction-cholestasis; *PFIC* Progressive familial intrahepatic cholestasis; *HSV* Herpes Simplex Virus; *CMV* Cytomegalovirus

### Clinical features and biochemical findings of DJS

Among the neonatal DJS infants, there were 2 males and 4 females (Table 2). All infants diagnosed with DJS were born full-term, and no infants showed failure to thrive (Table 3). All six showed jaundice and two had acholic stool. There were no infants with hepatomegaly or splenomegaly. All 6 patients had increased serum DB (median 7.4 mg/dL, range 3.7–11.0 mg/dL) and TB (median 11.5 mg/dL, range 5.8–15.6 mg/dL). The levels of AST (median 27 IU/L, range 25–61 IU/L) and ALT (median 15 IU/L, range 11–40 IU/L) were normal. Cholesterol (median 166 mg/dL, range 123–246 mg/dL), alkaline phosphatase (median 390 U/L, range 234–831 U/L ), and γ-glutamyltransferase (GGT, median 149 IU/L, range 33–209 IU/L) levels were normal or elevated. Prothrombin time (PT) was normal (median 1.1 international normalized ratio (INR)). Serum bile acid was measured in 1 patient and was over 150 µmol/L. Three infants underwent liver biopsy at 2 months of age and showed moderate intracanalicular and intracytoplasmic cholestasis but no melanin-like pigmentation, inflammation and fibrosis.

**Table 2:** Clinical features and biochemical findings of DJS

Patients number	Sex	Acholic stool	DB (mg/dL)	TB (mg/dL)	AST (IU/L)	ALT (IU/L)	Liver biopsy	Variant
1	F	-	4.1	5.8	25	14	Intracytoplasmic cholestasis Extramedullary hematopoiesis Suspicious bile duct loss	p.Arg768Trp
								p.Gly693Glu
2	F	-	11.0	15.6	61	15	-	c.2439 + 2T > C
								p.Tyr119SfsTer34
3	M	+	9.4	12.0	28	15	Intracytoplasmic cholestasis Extramedullary hematopoiesis	p.Arg768Trp
								p.Arg768Trp
4	M		7.0	13.4	25	17	-	p.Arg100Ter
								p.Arg1310Ter
5	F		3.7	8.1	54	40	-	p.Thr394Arg
								p.Asn718Ser
6	F	+	7.7	11.0	26	11	Intracanalicular and intracytoplasmic cholestasis Suspicious loss of interlobular bile ducts Degenerative change of interlobular bile ducts	p.Arg100Ter
								p.Arg768Trp

The laboratory results are the highest values ​​for the patients

**Table 3:** Comparison of Dubin Johnson syndrome and other causes of neonatal cholestasis

	Dubin Johnson syndrome (*n* = 06)	Other causes (*n* = 129)	*p* value
Male : Female	2 : 4	75 : 54	0.232
Term baby	6	98	0.173
Growth failure	0	21	0.284
Jaundice	6	81	0.125
Hepatomegaly	0	37	0.161
Splenomegaly	0	20	0.342
Acholic stool	2	46	0.908
DB (mg/dL)	7.4 (3.7–11.0)	4.7 (0.8–15.7)	**0.036**
TB (mg/dL)	11.5 (5.8–15.6)	7.6 (1.3–29.4)	**0.026**
Cholesterol (mg/dL)	166 (123–246)	154 (23–391)	0.467
AST (IU/L)	27 (25–61)	225 (17-3801)	**0.002**
ALT (IU/L)	15 (11–40)	143 (3-2493)	**0.007**
GGT (IU/L)	149 (33–209)	290 (11-1605)	0.265
PT (INR)	1.10 (0.91–1.11)	1.21 (0.77–3.28)	0.255

*DB* Direct bilirubin; *TB* Total bilirubin; *AST* Aspartate aminotransferase; *ALT* Alanine aminotransferase; *GGT* Gamma-glutamyl transferase; *PT* Prothrombin time

The laboratory results ​​are expressed as median (range)

### ABCC2 mutation analysis

One patient was homozygous for p.Arg768Trp and five patients were compound heterozygotes. A total of 8 different *ABCC2* variants were identified among the 12 alleles of neonatal DJS (Table 4). The most common variant was p.Arg768Trp (33.4%), followed by p. Arg100Ter (16.8%). Five mutations were known variants (p.Arg768Trp, p.Arg100Ter, c.2439 + 2T > C, p.Tyr119SfsTer34, and p.Arg1310Ter), and three were novel variants (p. Gly693Glu, p.Thr394Arg, and p.Asn718Ser). The five known mutations were all classified as pathogenic according to the ACMG standards / guidelines. *ABCC2* p.Gly693Glu and p. Thr394Arg were not observed among the normal population. *ABCC2* p.Asn718Ser showed a 0.06946% allele frequency in East Asians but was not found in non-East Asians. All of the novel variants were predicted to be most likely damaging through Polyphen-2. MutationTaster showed that all variants occurred in a well-conserved area and were classified as causing disease. According to the ACMG standards / guidelines, p.Gly693Glu was classified as likely pathogenic using multiple evidence categories: PM2, absent in the general population; PM3, *trans* with a pathogenic mutation; PP3, multiple computational evaluations support a deleterious effect; and PP4, patient phenotype highly specific for disease. The other two novel variants (p.Thr394Arg and p.Asn718Ser) were classified as VUS: PM2, absent in the general population; PP3, multiple computational evaluations support a deleterious effect; and PP4, patient phenotype highly specific for disease.

**Table 4:** Variants of *ABCC2* among infants with Dubin Johnson syndrome

Variant	Variant type	Allele frequency	Reported	ACMG classification	
c.2302C > T; p.Arg768Trp	Missense	4/12 (33.4%)	Known pathogenic	PS3, PM3, PM5, PP3, PP4	Pathogenic
c.298C > T; p.Arg100Ter	Nonsense	2/12 (16.8%)	Known pathogenic	PVS1, PM3, PP4	Pathogenic
c.2439 + 2T > C	Splice-site disruption	1/12 (8.3%)	Known pathogenic	PVS1, PS3, PM3, PP4	Pathogenic
c.351_355dup; p.Tyr119SfsTer34	Frameshift	1/12 (8.3%)	Known pathogenic	PVS1, PM2, PM3, PP4	Pathogenic
c.3928C > T; p.Arg1310Ter	Nonsense	1/12 (8.3%)	Known pathogenic	PVS1, PM3, PP4	Pathogenic
c.2078 g > A; p.Gly693Glu	Missense	1/12 (8.3%)	Novel	PM2, PM3, PP3, PP4	Likely pathogenic
c.1181C > G; p.Thr394Arg	Missense	1/12 (8.3%)	Novel	PM2, PP3, PP4	VUS
c.2153A > G; p.Asn718Ser	Missense	1/12 (8.3%)	Novel	PM2, PP3, PP4	VUS

*ACMG* American College of Medical Genetics and Genomics; *PVS* Pathogenic very strong; *PM* Pathogenic moderate; *PP* Pathogenic supporting; *VUS* Variants of uncertain significance

### Comparison of infants with neonatal Dubin-Johnson syndrome and infants with other causes of neonatal cholestasis

When comparing the clinical symptoms between neonatal DJS and infants with other causes of neonatal cholestasis, there were no differences in sex ratio, full term, jaundice, hepatomegaly, splenomegaly, and acholic stool (Table 4).

There were significant differences in AST and ALT levels when comparing neonatal DJS with infants with neonatal cholestasis from other causes. Levels of AST and ALT were significantly lower in infants with DJS (*p* = 0.002 and *p* = 0.007, respectively). There was no difference in levels of cholesterol, GGT, and in PT INR in either group (all *p* > 0.05), while DB and TB ​​were significantly higher in neonatal DJS infants (*p* = 0.036 and *p* = 0.026, respectively) than in infants with cholestasis from other causes.

Patients were followed up periodically with liver function tests for years (median 4 years, range 0.4–10.3 years). AST (median 29.5 IU/L, range 21–69 IU/L) and ALT (median 15.5 IU/L, range 6–27 IU/L) levels continued to be in the normal range. DB (median 1 mg/dL, range 0.6–2.1 mg/dL) and TB (median 1.6 mg/dL, range 1.2–3.7 mg/dL) gradually decreased but did not normalize.

## Discussion

A total of 60 different *ABCC2* gene mutations have been identified in infants with DJS through the HGMD, including missense, nonsense, frameshift, and splice-site disruption to date. All of our patients were confirmed to have neonatal DJS through genetic testing. Five mutations were known variants, and three novel variants were found in our study. The previously identified 5 variations are known to be pathogenic, which is consistent with the ACMG standards / guidelines: p.Arg768Trp (PS3, functional studies proven a deleterious effect; PM3, *trans* with a pathogenic mutation; PM5, new missense changes in amino acid residues with previously pathogenic changes; PP3, multiple computational evaluations support a deleterious effect; PP4, patient’s phenotype highly specific for disease;), p. Arg100Ter (PVS1, null variant; PM3, PP4), c.2439 + 2T > C (PVS1, PS3, PM3, PP4), p. Tyr119SfsTer34 (PVS1, PM2, PM3, PP4), and p.Arg1310Ter (PVS1, PM3, PP4). The p.Arg768Trp mutation in the nucleotide binding domain impairs the proper positioning and maturation of MRP2 to the apical membrane and induces neonatal cholestasis [11]. *ABCC2* c.2439 + 2T > C results in a splice site mutation at a conserved region, and functional testing confirmed that 168 nucleotides at nucleotide positions 2272–2439 were deleted [12].

The most common variant was p.Arg768Trp, followed by p.Arg100Ter. When examining the allele frequency of the gene in the general population through ExAC, p. Arg768Trp and p.Arg100Ter occupied 0.035% and 0.012% in East Asians, respectively, and 0.0053% and 0.0027%, respectively, in non-East Asians, suggesting that there is a difference in the frequency of the mutation in DJS infants of East Asians and non-East Asians [13]. These were the most common variants in Japanese DJS infants (both allele frequencies were 20%.) [5]. However, *ABCC2* gene mutations found in Korea and Japan were different from those reported in Taiwanese patients [1, 5].

There are studies on genotype-phenotype correlations in DJS [1, 5]. Lee et al. [1] reported that mutations involving the ATP binding cassettes (ABC) were associated with early-onset DJS, however, p.Thr394Arg found in this study was not involving ABC. In Japanese neonatal DJS patients, the mutation was either homozygous or compound heterozygous. These combinations were either 2 truncating mutations or a combination of truncating mutations and missense mutations [5]. It has been suggested that the combination of truncating and missense mutations may be a requirement for the phenotype of neonatal DJS [5]. However, our study has shown that neonatal DJS can occur by a combination of two missense mutations, such as homozygous p.Arg768Trp.

Novel variant, p.Gly693Glu (PM2, PM3, PP3, PP4) was classified as likely pathogenic using the ACMG standards/guidelines. The other two novel variants, p.Thr394Arg (PM2, PM3, PP4) and p.Asn718Ser (PM2, PM3, PP4) were classified as VUS. The ACMG standards / guidelines have established a new classification system for mutations that allows an indirect interpretation of whether novel variants are pathogenic mutations [7–10]. The VUS found in this study were predicted to be deleterious mutations by Polyphen-2 and MutationTaster. Functional studies are needed to understand the actual effects of these novel mutations.

Neonatal cholestasis occurs in approximately one in every 2,500 newborn babies [6]. The most common neonatal cholestasis is due to biliary atresia, accounting for 30% of all cases [6]. Genetic and metabolic causes account for approximately 10–20% of the total number of cases of neonatal cholestasis. The most common cause of neonatal cholestasis in premature infants is TPN [6, 14, 15]. Of our patients, infants diagnosed with biliary atresia accounted for 28% of the total number of cases of neonatal cholestasis diagnosed during the study period. In addition, TPN-induced cholestasis in our patients accounted for 4.5% of total number of cases. These incidences were similar to those published in previous papers [6, 14, 15].

As in Japanese studies, AST and ALT were within normal ranges in our patients [12]. In our study, we found that DB and TB were significantly higher than neonatal cholestasis due to other causes.

In liver biopsies of adult patients with DJS, melanin-like pigmentation deposited in hepatocytes is generally found. In some cases, the liver appears as a black liver [16]. It is known that cholestasis occurs due to abnormal MRP2 function. [17–19]. This protein actively transfers bilirubin from the hepatocyte to the bile duct. Mutations in the *ABCC2* gene cause abnormalities in the function of MRP2, resulting in cholestasis and black liver as a result of the failure to release bilirubin and bile into the bile ducts [5]. However, melanin-like pigmentation was found in only 38% (3 of 8) of the neonatal DJS patients who underwent liver biopsy [5]. In our study, liver biopsy revealed no evidence of melanin-like pigmentation. Therefore, unlike in adult DJS, it may be difficult to identify characteristic findings of DJS through liver biopsy in neonatal DJS.

In infants with DJS, the symptoms of neonatal cholestasis may be more severe due to gene mutations of *ABCC2* as well as the immaturity of the physiological metabolism of bile [1]. As the maturity of the liver gradually increases, the degree of cholestasis gradually improves, and the patients become asymptomatic. Cholestasis may reappear in adulthood due to drug, hormonal changes, infection, and pregnancy.

Long-term follow-up studies of neonatal DJS have also addressed the biphasic appearance of cholestasis [1]. In this study, hyperbilirubinemia decreased with age. The infants were jaundiced only during the neonatal period. Therefore, failure to diagnose DJS early in the neonatal period may delay the diagnosis. Considering the biphasic appearance of DJS, some may be diagnosed in the adulthood. Early diagnosis through neonatal cholestasis gene panel can prevent unnecessary evaluation. It is very important that the differential diagnosis for neonatal cholestasis with normal liver parameters except for TB/DB includes DJS. Apart from the gene panel which can test for DJS, the urinary coproporphyrin isomer I composition will help differentiate DJS as well [20].

## Conclusions

This is the first study to analyze *ABCC2* gene mutations in Korea. DJS accounted for 4.5% of all cases of neonatal cholestasis, and three novel variants were identified. The neonatal cholestasis gene panel is useful in diagnosing DJS. When AST and ALT levels are normal in infants with neonatal cholestasis, genetic testing for DJS should be considered.

## Data Availability

The raw data analysed during the current study are not publicly available due to the aim to protect the confidentiality of the patients but are available from the corresponding author on reasonable request.

## Abbreviations

ACMG: American College of Medical Genetics and Genomics
*ABCC2*: Adenosine triphosphate-binding cassette subfamily C member 2
DB: Direct bilirubin
DJS: Dubin-Johnson syndrome
GGT: γ-glutamyltransferase
TB: Total bilirubin
TPN: Total parenteral nutrition
VUS: Variants of unknown significance
